# Proteomic methods for the study of porcine acute phase proteins – anything new to detect?

**DOI:** 10.1007/s11259-023-10170-6

**Published:** 2023-07-15

**Authors:** Ingrid Miller, Elisabetta Gianazza

**Affiliations:** 1 Institut für Medizinische Biochemie, Veterinärmedizinische Universität Wien, Veterinärplatz 1, A-1210 Wien, Austria; 2 Dipartimento di Scienze Farmacologiche e Biomolecolari, Università degli Studi di Milano, Via Balzaretti 9, I-20133 Milano, Italy

**Keywords:** Acute phase proteins, Inflammation, Proteomics, Pig

## Abstract

Acute phase proteins (APPs) reflect the health status of individuals and are important tools in diagnostics, as their altered levels are a sign of disturbed homeostasis. While, in most cases, quantitation of known serum APPs is routinely performed by immunoassays, proteomics is helpful in discovery of new biomarker candidates, especially in samples other than body fluids. Besides putting APP regulation into an overall context of differentially abundant proteins, this approach can detect further details or outright new features in protein structure or specific modifications, and help understand better their function. Thus, it can show up ways to make present diagnostic assays more sensitive and/or specific, or correlate regulations of disease-specific proteins. The APP repertoire is dependent on the species. The pig is both, an important farm animal and a model animal for human diseases, due to similarities in physiology. Besides reviewing existing literature, yet unpublished examples for two-dimensional electrophoresis in connection with pig APPs highlight some of the benefits of proteomics. Of further help would be the emerging targeted proteomics, offering the possibility to determine particular isoforms or proteoforms, without the need of specific antibodies, but this method is presently scarcely used in veterinary medicine.

## Introduction

The acute phase response (APR) is an early defence system of the organism against a variety of noxa, before specific adaptive or immunologic reactions can be activated or developed. One of the key features is the up- or, less frequently, the down-regulation of the synthesis by the liver of a number of plasma proteins. The quali- and quantitative data about these acute phase proteins (APPs) lack the specificity to identify any specific stressful / traumatic / inflammatory condition but, as key markers of the adaptive reaction, they do contribute to its recognition and are then extremely useful for monitoring its course (Whicher and Westacott 1992; Murata et al. 2004; Cray et al. 2009).

While all mammals share the ability to raise an acute-phase reaction, the details of the process differ from one species to another. In particular, these differences were associated with the APPs identified and/or their abundance, with the time-course of the reaction, and with the changes in protein post-translational modifications (PTMs).

In this review, we deal with the APPs in pigs in the field of proteomics. With more than 700 million heads in 2022, accounting for a 35% share in meat production, this livestock has top relevance for the farming industry (https://www.statista.com/statistics/263964/number-of-pigs-in-selected-countries/, https://www.statista.com/statistics/237632/production-of-meat-worldwide-since-1990/). In a completely different yet crucial perspective, pigs and minipigs are sometimes used as laboratory animals, mainly while setting up surgical procedures (including organ transplants) and following up pharmacological treatments. In connection with either area of interest, checking for wellbeing in production animals under different breeding and feeding conditions, recognizing the occurrence of distress / disease, monitoring the outcome of surgical or pharmacological interventions, all may benefit from the assessment of APPs (Petersen et al. 2004; Klauke et al. 2013b).

## APPs in pig and methods of their determination

### Pig APPs

Similar to most other mammals, also in pig the main positive APPs (upregulated in inflammation) are C-reactive protein (CRP), serum amyloid A (SAA) and haptoglobin (HPT) (Heegaard et al. 1998; Eckersall et al. 1999). In addition, particular for pig is a marked upregulation of inter-α-trypsin inhibitor heavy chain H4 (ITIH4), formerly called major acute phase protein (pig-MAP) (González-Ramón et al. 1995). In the following, we will use either names, pig-MAP or ITIH4, in accordance to the names used in the respective references. Negative acute phase proteins are apolipoprotein A-I (apo A-I) and albumin, at least in chronic inflammation (Heegaard et al. 2011). Some other serum proteins reported as regulated in APR under distinct pathologic or experimental conditions are compiled in Table 1. The degree of regulation has been found to differ depending on the type of infection agent or external influence (transport, housing, feeding) or physiological state (weaning, pregnancy, farrowing) (Gómez-Laguna et al. 2011; Heegaard et al. 2011).

**Table 1:** Main pig APPs

Direction of concentration change	APP name	References
positive APPs	CRP, SAA, HPT, ITIH4	(Heegaard et al. 1998), (Eckersall et al. 1999)
	ceruloplasmin	(Eckersall et al. 1996)
	fibrinogen (plasma)	(Sorrells et al. 2007)
negative APPs	apo A-I, albumin	(Heegaard et al. 2011)
	transthyretin	(Campbell et al. 2005)
variable	α_1_-acid glycoprotein (AGP)	(Eckersall et al. 1999), (Heegaard et al. 2013)

**Fig. 1:**
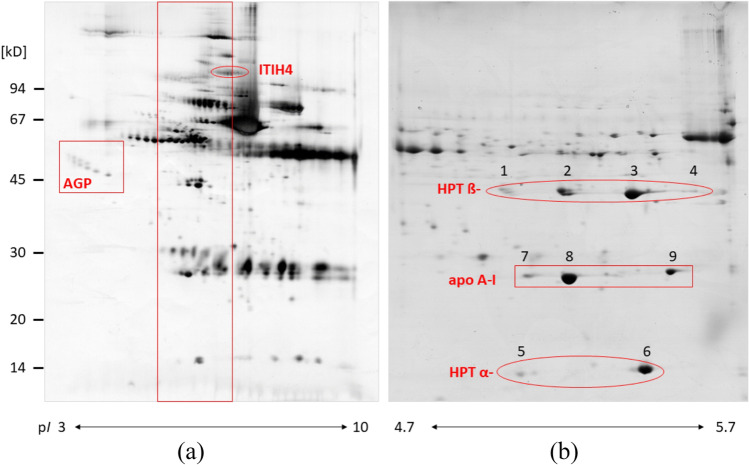
2-DE of pig serum in different pH-ranges. IPG in (**a**) pH 3-10 nonlinear; in (**b**) pH 4.7-5.7. Stains: (a) silver stain; (b) colloidal Coomassie Brilliant Blue G-250. Animals: (a): clinically healthy adult animal; (b): animal with inflammation (infection with *E. coli*). Small box in (a) indicates position of AGP and corresponds to the images in Figs 3, 4, 5. Big box indicates the region displayed in (b). The positions of haptoglobin (HPT) α- and ß-chains and of the apolipoprotein (apo) A-I spots are indicated (spot numbers as used in manuscript text). Oval encircles ITIH4, another important positive APP in the pig

APPs, though acting as a first line of defence, are very different in nature: for instance, CRP and SAA are of low serum concentration in healthy animals, but very fast-reacting and increasing upon challenge. The homopentameric CRP promotes agglutination, bacterial capsular swelling, phagocytosis and complement fixation through its calcium-dependent binding to phosphorylcholine. It can interact with DNA and histones and may scavenge nuclear material released from damaged circulating cells (Lim et al. 2001). SAA, in contrast, is a small apolipoprotein of the HDL complex, able to directly induce pro-inflammatory cytokines and chemokines, and it plays a role in cell-cell communication, though not all its functions are completely clear yet (Sack 2018). In contrast, HPT, apo A-I and albumin are moderate to major serum proteins, well visible in the protein pattern both in healthy and in pathological states (e.g. in two-dimensional electrophoresis, Fig. 1a). HPT, a tetramer of two alpha and two beta chains, captures free plasma hemoglobin to allow heme iron recycling and acts as an antioxidant (Naryzny and Legina 2021). ITIH4 (indicated also in Fig. 1a) inhibits serin endopeptidases and may play a role not only in APR but also in liver development and regeneration (Gonzalez-Ramon et al. 2000). Table 1 details their type of regulation (up, down).

### APPs in serum

#### Quantitation by non-proteomic methods

A large share of the literature data about pig APPs is a quantitative estimate as the concentration of individual proteins was measured tens of times in biological fluids – most often in plasma/serum, but also in saliva and in meat juice. The number of proteins tested in each investigation varied from 1 (e.g. SAA (Williams et al. 2009)) to 8 (albumin, α-fetoprotein, AGP, fetuin, HPT, α_1_-protease inhibitor, transferrin, and pig-MAP *plus* IgG (Martin et al. 2005)). Measurements were made under baseline conditions (AGP, HPT, pig-MAP, SAA, and transthyretin in swine from commercial farms (Clapperton et al. 2007), HPT and pig-MAP in two swine breeds (Piñeiro et al. 2009c)), while investigating also the influence of age (Martin et al. 2005; Christoffersen et al. 2015) and sex (Christoffersen et al. 2015) on the reference protein levels. Measurements were also made after experimental inflammation, induced either by subcutaneous injection of turpentine (albumin, AGP, apo A-I, α_2_-macroglobulin, CRP, fetuin, HPT, pig-MAP, α_1_-protease inhibitor, transferrin (Lampreave et al. 1994), AGP, ceruloplasmin, CRP, HPT (Eckersall et al. 1996)) or by lipopolysaccharide (LPS) administration (ceruloplasmin, CRP, HPT (Frank et al. 2005), SAA (Williams et al. 2009)).

On the evidence of a concordance between animal health / welfare and carcass quality in pork production chains (Klauke et al. 2013b), the effect of several breeding parameters has been assessed through the measurement of APPs: two such parameters were, for instance, feeding (e.g. pig-MAP decreased as the protein content of the diet increased (Hermes et al. 2009)) and housing (AGP, fibrinogen, HPT changed little between gilts housed in small groups in pens and gilts housed in standard industry stalls (Sorrells et al. 2007)). The largest number of investigations, however, has been devoted to diseases, mainly viral, bacterial and nematode infections, under both, field and experimental conditions (just an example with the definition of CRP-apo A-I-MAP or CRP-apo A-I-HPT as optimal diagnostic APP combinations (Heegaard et al. 2011)).

Quantitation of pig APPs was most often carried out through immunological assays. Some of the former data were produced by radial immunodiffusion (Lampreave et al. 1994); most of the recent ones are obtained by ELISA, a routine procedure for which a number of commercial kits are available. Research papers dealing with the setup, standardization or validation of such assays are, for instance: on sandwich ELISA for CRP and SAA (Tecles et al. 2007), and for pig-MAP (Piñeiro et al. 2009b); on direct non-competitive ELISA for pig-MAP (Tecles et al. 2007); on competitive ELISA for SAA (Soler et al. 2011). Alternatively, an immunofluorometric assay for CRP was proposed (Martinez-Subiela et al. 2007) and an immunochromatographic method allowing the recognition of elevated levels of pig-MAP was reported (Piñeiro et al. 2013). For APPs with enzymatic activity, also functional assays have been used: this includes evaluating by spectrophotometric methods the peroxidase activity of haptoglobin-hemoglobin complexes (Tecles et al. 2007) as well as the oxidase activity of ceruloplasmin (Martínez-Subiela et al. 2007).

Standardization of pig APP testing has long been a concern (Eckersall et al. 1999; Skinner 2001). Indeed, to ensure that results obtained in the laboratory or on the farm are comparable and of consistent quality, the calibration of assay methods needs to be harmonized. In February 2000 the European Commission Directorate General Research Concerted Action was established to fulfil the task of international standardization of APP.

#### Gel-based proteomics (2-DE)

Gel-based methods have a long tradition in the investigation of the serum protein repertoire and were the first ones to apply in this topic. Several experiments to characterize the main components of pig plasma/serum proteome after in-gel separation have been carried out using the two-dimensional gel format. Some of them were undertaken even before the term “proteomics” was coined: one significant example resorted to crossed–affinoimmunoelectrophoresis (namely, zonal electrophoresis in agarose containing concanavalin A (Con A), followed by migration at right angle in an antibody-containing agarose) (Lampreave et al. 1993). The majority of investigations involved two-dimensional electrophoresis (2-DE) according to either the IPG-DALT or the DIGE (Marco-Ramell et al. 2011; Yang et al. 2012; Marco-Ramell et al. 2016) protocol (with a combination of DIGE and iTRAQ (Marco-Ramell et al. 2016)).

The reference publication for this research field is the one by Miller *et al.* (Miller et al. 2009). The paper features a 2-DE map of serum collected from healthy male pigs under baseline conditions, in which 26 proteins are identified across 39 spots. More on the findings by this investigation and additional data using the same methodology are reported in the section “Benefits of gel-based proteomics”.

Dealing with physiological aspects in the animal’s life, some investigations are devoted to changes in proteome, including the relative abundance of APPs, along the developmental stages. The earliest such study focused on the glycosylation of proteins from 26-day-old porcine fetuses to birth, and in adult pigs; some proteins, such as transferrin and α-fetoprotein, are Con A-reactive during the entire developmental period; for others, like AGP, the ratio between non-reactive and reactive varies with time (Lampreave et al. 1993). Moving to postnatal life, an investigation addressing the 12 to 30 weeks period concentrated on two spots, differentially regulated in the growth stages; one of them was identified as haptoglobin α_1_S, the other as apolipoprotein A-IV (APOA4): with time, the concentration of the former was decreasing, that of the latter was increasing (Chung et al. 2008). A later work from the same research group extended the description of the time-course concentrations to nine proteins: immunoglobulin kappa, lambda, and gamma, retinol-binding protein, albumin, fibrinogen alpha and gamma, antithrombin, and α_1_-antitrypsin (Chung 2011). The latest and most comprehensive of such investigations characterized as many as 37 proteins as differentially regulated in pigs from birth to 150 days of age (Srikanth et al. 2017).

A typical way to induce an APR in a controlled way is by injection of LPS. Two reports address this aspect by analyzing the proteome at different time points after trigger. One of them samples at 6 hours post-injection (15 μg/kg); while the qualitative changes in the pattern are made evident by 2-DE, the actual quantitation is carried out by ELISA. CRP and HPT turn out to be the fastest reactive APP, while no differences can be observed at the selected early time point in the levels of pig-MAP and transthyretin (Yin et al. 2017). Another report samples at 24 hours post-injection. At this later time point the changes involve a much higher number of APPs: serum albumin, transferrin, light and heavy immunoglobulin chains, and major acute phase proteins including HPT, SAA2, CRP, AGP, β_2_-glycoprotein 1, α_2_-HS-glycoprotein, and α_1_-antitrypsin (Olumee-Shabon et al. 2020).

To evaluate the effect of breeding conditions on animals’ wellbeing, the level of APPs was evaluated in pig serum in connection with housing (Marco-Ramell et al. 2011; Marco-Ramell et al. 2016) and feeding parameters (Herosimczyk et al. 2015; Grubbs et al. 2016). High-density (0.25 m^2^/pig) housing resulted in a significant increase of pig-MAP, but no changes were observed in serum cortisol or other acute phase proteins in comparison with low-density (0.50 m^2^/pig) housing (Marco-Ramell et al. 2011). However, housing of gilts in individual stalls increased overall stress markers including altered levels of HPT, apo A-I and α_1_-antichymotrypsin 3 (Marco-Ramell et al. 2016). Pigs that consume less feed than expected have a lower residual feed intake (RFI), are more feed efficient, and they are therefore economically better for lean production compared to pigs with high RFI. Gelsolin, vitronectin, and serine protease inhibitor A3 (serpinA3) were found at significantly higher concentrations in the serum of the more efficient low-RFI pigs (Grubbs et al. 2016). Addition of water extract of inulin-type fructans to the diet of growing piglets resulted in increased levels of fibrinogen along with decreased levels of plasminogen thus shifting the pro- and anti-coagulation balance (Herosimczyk et al. 2015).

Moving to overt pathology, APPs were assessed in wasting (Yamane et al. 2006) as well as in non-infectious growth-rate retardation (Gutiérrez et al. 2019), but the largest number of investigations was devoted to infections. Already the report by Miller *et al.* (Miller et al. 2009) addressed the effect of such conditions: after viral infection (PCV2), pig-MAP, HPT, hemopexin and all types of immunoglobulins are found upregulated, while α1B-glycoprotein and α_2_-HS-glycoprotein are downregulated; after bacterial infection (*Actinobacillus pleuropneumoniae, A.pp.*), the levels of pig-MAP, HPT, IgM, and IgG increase.

Viral infection was then specifically addressed for classical swine fever virus (CSFV) (Sun et al. 2011) and for highly-pathologic porcine reproductive and respiratory syndrome virus (HP-PRRSV) HuN4 (Yang et al. 2012). While the diseases brought up by the above pathogens are multisystemic, the conditions investigated in connection with bacterial infection are mainly targeting the gastrointestinal tract: among them peritonitis (Thongboonkerd et al. 2009) and necrotizing enterocolitis (Jiang et al. 2020). One of the areas of interest is in the handling of preterm newborn piglets (Muk et al. 2019; Jiang et al. 2020). The effects of antibiotics to reduce inflammation and decrease APP level while counteracting bacterial infection has also been investigated (Jiang et al. 2012; Soler et al. 2016).

As the standard way to the identification of partially or fully resolved protein components, MS procedures are an essential part of all proteomic protocols. Reliability of identification on the basis of MS results rests in turn on the availability of a comprehensive species-specific database of protein sequences to be taken as reference. For swine, one such resource is the Pig PeptideAtlas (Hesselager et al. 2016), which contains data from 25 tissues and 3 body fluids (plasma, colostrum and synovial fluid), mapped to as many as over 7000 proteins. The number of protein-coding genes in pig genome is estimated to be approx. 10 times higher than that (https://www.ncbi.nlm.nih.gov/genome?term=sus%20scrofa%20%5BOrganism%5D&cmd=DetailsSearch); identification of proteins outside the set covered by the Atlas may still be possible, as usual, by taking advantage of the homology to components in different, fully characterized species (Cottrell 2011).

#### Gel-free proteomics (LC-MS/MS)

A few of the reports dealing with pig plasma/serum composition use MS techniques alone, while exploiting their potential for both qualitative and quantitative evaluations. A remarkable example of the first type of such investigations is provided by the comprehensive analysis of swine plasma proteome (Tu et al. 2011). In this study, combinatorial peptide ligand library (CPLL) treatment was used to reduce the protein concentration dynamic range, achieving a >100-fold enrichment of the lower abundance proteins. Digestion with two proteolytic enzymes (trypsin and GluC) and complex LC-MS-analysis resulted in the identification of over 3400 proteins, spanning a concentration range of 9-10 orders of magnitude. Together, these data may define the reference pattern of pig plasma composition.

One report using the MS approach has investigated the acute phase response as induced by one of the standard inflammatory stimuli, namely turpentine injection (López-Martínez et al. 2022). Six hours after the challenge, 26 proteins were found at significantly different circulating levels; GO enrichment analysis showed significant over-representation of the following terms: antimicrobial humoral response, focal adhesion assembly, and serine-type endopeptidase activity.

Some more reports have instead dealt with specific pathological conditions: these conditions included bacterial (Muk et al. 2019) and viral (Genini et al. 2008; Liu et al. 2011; Genini et al. 2012; Koene et al. 2012) infections; trauma as hypoxia-ischemia (Kyng et al. 2018), hemorrhagic shock, tissue injury, liver reperfusion, hypothermia, and comminuted bone fracture (Cudjoe et al. 2019)), and diet-induced metabolic syndrome (Bell et al. 2010; Pas et al. 2018). The analytical approach was usually LC-MS/MS, but it was SELDI-TOF in a few cases (Genini et al. 2008; Genini et al. 2012; Koene et al. 2012).

A couple of studies have been devoted to a single APP, the SAA protein and its isoforms. These were investigated in serum samples from pigs experimentally infected with *Staphylococcus aureus,* using a Selected Reaction Monitoring (SRM) approach as a quantitative targeted MS method (Leuchsenring et al. 2020). SAA2 was found to be the dominating circulating isoform under baseline conditions, while during the acute phase response to infection, SAA2, SAA3 and SAA4 increased approx. 10, 15 and 2 times, respectively. LC-MS/MS was also used to analyze the amyloid material extracted from the spleen of pigs after *Streptococcus suis* infection; besides SAA2 fragments, a unique amyloid sequence was identified in this specimen (Kamiie et al. 2017).

### APPs in other sample types

#### APPs in saliva

Saliva has recently become a specimen of interest in medicine, as samples are obtainable in a simple, non-invasive and relatedly stress-free way, also in animals. From its protein composition there is an overlap between saliva-specific proteins secreted by salivary glands and proteins also found in serum, although protein concentrations in saliva are about a factor of 20 lower than in serum (Dawes 1972). Some of the main APPs known for serum, CRP and HPT, have been found similarly regulated when comparing saliva and serum, though they appear in saliva at more than thousand-fold lower concentrations (Sánchez et al. 2021). For their evaluation, specific time-resolved immunofluorometry assays were developed and APP profiles of the analytes determined in time-course studies in the two matrices in parallel (Gutiérrez et al. 2012). It is interesting to note that not only age (Gutiérrez et al. 2009; Gutiérrez et al. 2013b) but also the circadian rhythm influences some of their levels. Indeed, HPT levels were approx. twice as high in the morning in comparison with late afternoon, while CRP levels stayed invariant (Gutiérrez et al. 2013b).

After setting up a reference proteome pattern in 2-DE (Gutiérrez et al. 2011), proteome changes of porcine saliva were investigated in animal groups in different conditions, e.g. systemic disease (Gutiérrez et al. 2013a). Among the differentially regulated spots subjected to MS for identification, also spots from the HPT β-chains were found which correlated nicely to immunoassay results of a previous health check in these animals. Besides this already known APP, salivary lipocalin, lipocalin 1, double headed protease inhibitor protein, adenosine deaminase, three proteins of the S100 family and pancreatic α-amylase were picked up as differentially abundant and of potential interest for this sample type. One of them, adenosine deaminase (ADA), was further followed in a specific assay and reported as a new potential salivary marker (Gutiérrez et al. 2017), confirmed by the study of another group (Sali et al. 2021).

#### APPs in meat juice

Meat juice is an alternative specimen in which pig APPs are sometimes evaluated, if it comes to meat quality. Meat juice is obtained by collecting the dripping from frozen flesh samples once thawed; harvesting is done after placing the meat piece either over a test tube or inside a clamped plastic bag. Meat juice composition has never been investigated in detail by proteomic procedures; only one, very old, report deals with electrophoresis of juice obtained by pressure from *musculi adductores* (Scharner and Schürer 1974). Meat juice is used as a diagnostic specimen in ELISA tests for antibodies against pathogens causing both zoonoses and production diseases (Meemken et al. 2014); for high sample throughput, such a multi-parameter testing may be carried out with miniaturized protein microarrays (Loreck et al. 2020). While far less specific than serology, evaluation of APPs during routine veterinary inspection at abattoirs is broader in scope. Typically, in such screenings CRP and HPT are tested; sensitivity of these proteins together to detect animals with organ alterations is 86% (Gutiérrez et al. 2015b). Receiver Operating Characteristic (ROC) analysis shows the highest sensitivity-specificity pairs, nearly 80-90 percent, at cut-off levels of 83 and 10 μg/ml for HPT and CRP determinations (Gutiérrez et al. 2015a). APP concentrations in meat juice closely correlate to those in plasma (Piñeiro et al. 2009a). As an alternative to ELISA, a (surface acoustic wave) sensor chip with specific antibodies has been proposed to measure HPT concentration (Klauke et al. 2013a).

#### APPs in other body fluids or tissue

As inflammation and therefore also APR is an integral part of almost all types of disease, APPs are likely to be picked up in discovery proteomics when samples from healthy and disease status are differentially compared in search for specific biomarker candidates. For instance, HPT was picked up in an iTRAQ study among the 20 proteins with the highest fold changes in synovial fluid in a porcine model of injury-induced posttraumatic osteoarthritis (Kiapour et al. 2019). It was also detected in the porcine genital tract of healthy females and its concentration was followed in different oestrous stages. Results suggested that it plays an important role in the reproduction process and its addition during *in vitro* embryo production improved the blastocyst rates (García-Vázquez et al. 2021). Among the 53 differentially abundant proteins in jejunum mucosa of heat-stressed finishing pigs, 10 proteins were attributed to stress and defence response, including upregulated HPT and decreased albumin (Cui and Gu 2015). Infection with porcine circovirus 3 (PCV3) affected the lungs of specific pathogen-free piglets, highlighting changes in metabolic processes, innate immune response, MHC-I and MHC-II components, and phagosome pathways primarily, as determined in a study through an iTRAQ approach (Jiang et al. 2020). One group of proteins which increased almost 2-3 fold were APPs, namely HPT, ITIH4, SAA, clusterin, and AGP, likely a sign of severe lung inflammatory responses. Sometimes, changes in the “classical” APPs are hidden in long lists of differentially regulated proteins in supplemental files (e.g. the exposure of tracheal proteins to ammonia (Wang et al. 2021)).

However, proteomic investigations of tissue or body fluids in pathological states or disease are still underrepresented for farm animals. More detailed investigations using the comprehensive approach of the proteomic perspective may yield tissue-specific markers of inflammation – whether newly detected or validated from previous evidence.

The pig is a non-primate mammal that closely resembles man in anatomy, physiology and genetics. Thus, pigs are sometimes taken as animal models for biomedical research on human conditions, and were also a topic for proteomic studies (Bendixen et al. 2010; Verma et al. 2011; Bassols et al. 2014). Examples of investigations in which the levels of circulating APPs were evaluated are ITIH4 in hepatocellular carcinoma with non-alcoholic fatty liver disease (Nakamura et al. 2019) and SAA, HPT, and CRP during the repair of experimentally induced articular cartilage (Tothova et al. 2019). The latter study applied specific APP assays.

## What proteomics can do in addition

This section deals with the benefits of proteomics and related omics beyond discovery of potential markers or quantification of a larger panel of proteins, namely the characterization of the components of interest – often a few protein species or proteoforms, as a result of differential post-translational modifications (PTMs) or truncation/aggregation. Learning more about the affected proteins and protein species will help to develop better methods for the detection of potentially useful markers - in the specific case, of pig APPs.

### The benefits of gel-based proteomics

In all proteomics methods, appropriately solubilized samples first undergo a separation step followed by quantification and identification. In the case of gel-based proteomics, proteins are separated and the two-dimensional pattern revealed by staining (by fluorophores with labelling samples before separation like in DIGE or after separation, e.g., with SYPRO stains; by colorimetric stains like Coomassie Brilliant Blue or silver (Miller et al. 2006)) and spot volumes corresponding to quantities measured. What makes 2-DE gels specific is the fact that minor changes in the amino acid backbone or changes in the side chains or ligands are clearly visible as pattern changes, provided that those modifications change the isoelectric point and/or the size of the molecule.

For gel-free proteomics, proteins are digested into peptides, most often with the enzyme trypsin. Peptides are then separated by chromatography and their size and breakdown pattern evaluated by mass spectrometry. Proteins are identified with dedicated software which can re-assemble related peptides into their original proteins, based on searches in specific protein databases. The sequence coverage for these protein identifications is seldom complete and the setup only allows detection of peptides either without modifications or with known ones. Thus, other changes or even protein truncations may go undetected, although they were of physiological importance (Marcus et al. 2020).

The pros and cons of both proteomics approaches have been discussed in literature and there are several recent papers highlighting the benefits of gel-based proteomics and contradicting current trends regarding gels as outdated (Rogowska-Wrzesinska et al. 2013; Carbonara et al. 2021; Ercan et al. 2023).

In the following sections, most examples are taken from gel-based studies using two-dimensional electrophoresis, where studies of this kind have been performed earlier and where changes in the protein patterns are easier to see.

### Protein characterization (as a basis for specific tests)

The 2-DE map with identification of the main porcine serum proteins in healthy animals has been published over a decade ago (Miller et al. 2009). The paper contains also the gel image of serum from a pig with very severe infection; marked changes are observed in the intensity of several spots/spot rows: these mainly correspond to APPs but involve as well proteins related to immune response. Figure 1 displays how the resolution in the 2-DE gel of porcine serum can be increased by using as a first dimension a home-made IPG spanning just one pH-unit (Fig. 1b) instead of the full pH-range 3-10 (Fig. 1a). In the latter case, serum from an infected animal was separated, showing the pH-region of interest for closer investigation of the APPs HPT and apo A-I. HPT is visible as two spot chains, α and β, both consisting of at least two spots of similar molecular mass, but different p*I*. A previous study has evaluated the differences between the individual spots of the HPT chains (Marco-Ramell et al. 2014). N- and O-glycosylation contribute to the heterogeneity of the β-chain, which has four potential glycosylation sites. O-Glycosylation was unexpected, as this type of modification does not occur in the homologous human protein of healthy individuals. The α-chain is not glycosylated, but spot #5 may represent an oxidized form of spot #6. In severe inflammation (infection with porcine circovirus 2, PCV2), the overall concentration of HPT increased to about 1.5-fold in the reported animal group (Marco-Ramell et al. 2014). When evaluating the single spots of the HPT β-chain in Coomassie-stained 2-DE zoom gels similar to the one shown in Fig. 1b, spot volume ratios stayed constant. This is supported by glycan analysis, finding no differences in the glycosylation pattern of healthy animals. We have found similar results when investigating serum samples from an infection study with *A.pp.* (animal experiment (Menzel et al. 2014); 2-DE results unpublished). This leads to the conclusion that assays detecting only some of the proteoforms (protein spots) will still reflect the overall regulation of haptoglobin, however, potentially with a lower sensitivity.

**Fig. 2:**
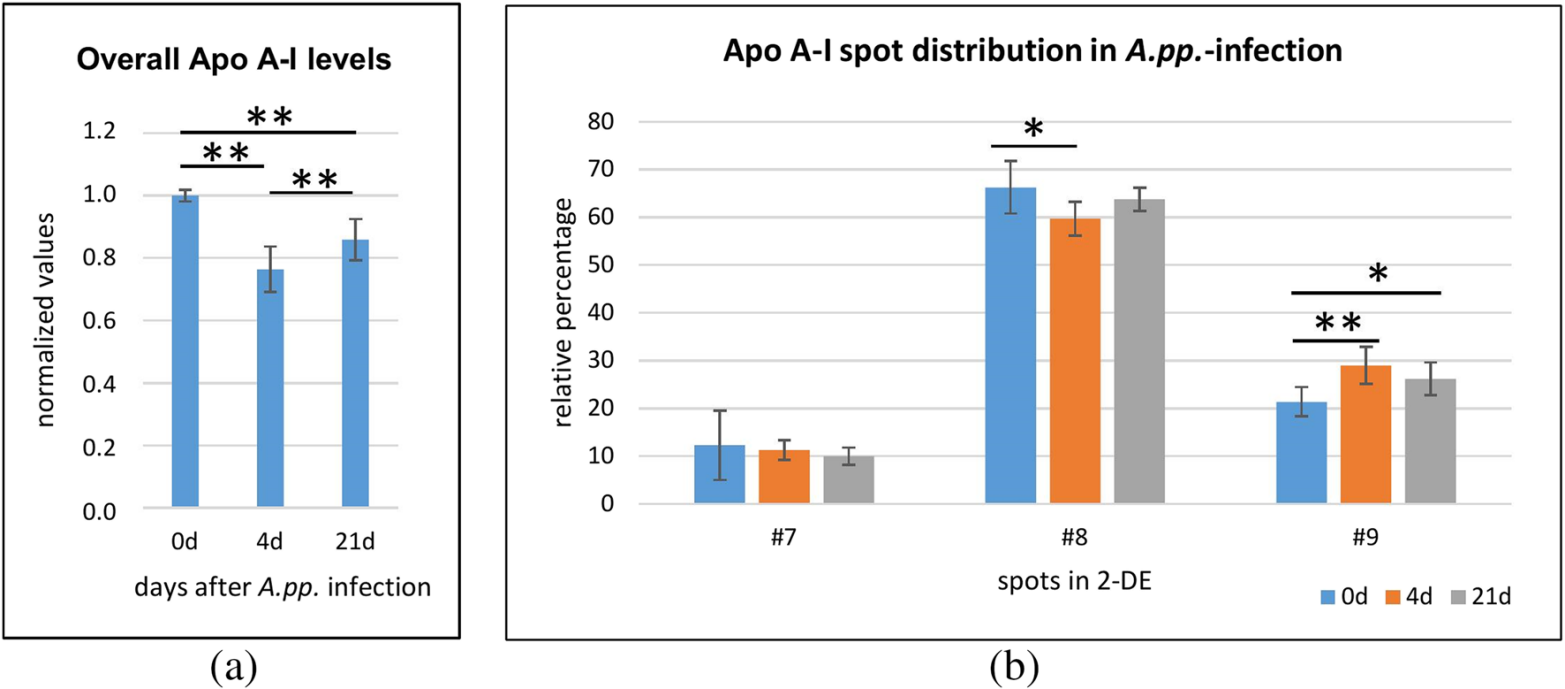
Regulation of apo A-I. (**a**) Apo A-I regulation in course of an infection with *A.pp*. (control on d0, d4 and d21 post-infection); evaluation: band intensity in SDS-PAGE with Coomassie Brilliant Blue stain, normalized onto respective band in controls (*n*=4). (**b**) Relative percentages of apo A-I single spots in a 2-DE gel stained with Coomassie Brilliant Blue, same time course and samples as in (a) (*n*=4). Spot numbering as in Fig. 1b. Statistical significance: * *p*<0.1; ** *p*<0.05

The second APP prominently visible in Fig. 1b is apo A-I, a well-known negative acute phase protein in pig and other mammals. A study devoted to its heterogeneity applied an approach similar to the one previously described for HPT (Marco-Ramell et al. 2015). This protein is not glycosylated; its main spot #8 represents the mature protein, while spot #9 corresponds to the protein precursor (the propeptide was detected by MS-analysis). In the PCV2 infection described above, overall apo A-I concentration dropped to about 80%. At the same time, the relative ratio between spots #7 and #8 dropped, but the one with #9 increased, with a statistical significance of p≤ 0.001. This may be related to a lower rate of protein maturation and changes in lipid metabolism, as hypothesized in the respective paper. On the analytical scale, it may mean that this unbalanced alteration in spot intensity is a chance for developing assays specifically targeted to spot #8 or #9, differentiating between mature form and precursor molecule. A slightly less extensive change was reported by the same authors in an infection with *S. typhimurium*, while the effects of PRRS virus and *E. coli* were not as pronounced (Marco-Ramell et al. 2015). We found a similar disbalance in spot regulation also in the samples of the above-mentioned *A.pp.* experiment (Fig. 2) and hypothesize that this may be a sign of very severe infections, independently of the bacterial or viral agent. It is interesting to note that in the time course of the inflammatory response changes in both apo A-I concentration and relative spot ratios are most extensive at day 4, whereas on day 21 both parameters are going back to levels closer to baseline.

### Protein pattern changes due to testing conditions and animal age

**Fig. 3:**
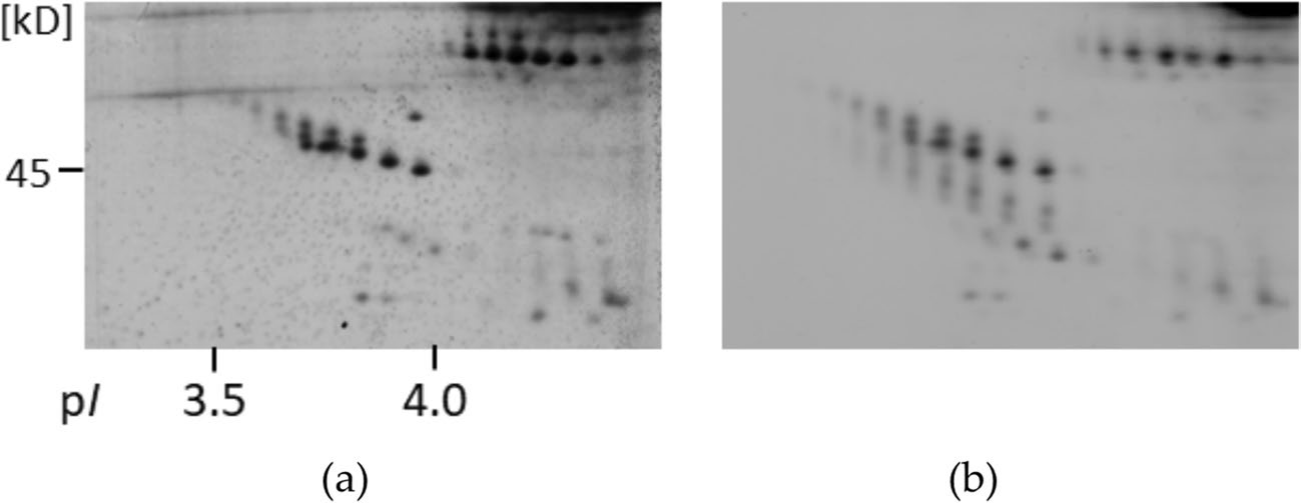
AGP and the influence of staining. 2-DE pattern of serum from an adult pig in a zoom gel with an IPG pH-range 2.5-5; only detail shown (corresponding to region of the small box in Fig. 1a). Comparison of detection methods: (**a**), silver stain; (**b**), Cy2-labelled sample and extraction of fluorescent pattern as grey scale; in both cases serum of the same animal was used and the two samples were run side by side in one gel

AGP is a very acidic protein of about 43 kDa. In pig it is reported as positive or negative APP, or even as unchanged in APR, depending on the type of infection (see Table 1), but maybe also on the test system. In 2-DE, porcine AGP is best resolved when using as a first dimension home-made IPGs of pH range 2.5-5 (Miller et al. 2009); this protocol allows the separation of two distinct if closely spaced spot chains when investigating adult pig serum (Fig. 3a). The pattern is characteristic for classical (reducing, denaturing) 2-DE, but changes when omitting reduction or using completely native electrophoresis conditions (Heegaard et al. 2013). This phenomenon is well-known also for other proteins and can prove helpful for further protein characterization (Miller et al. 2010). In the case of (Heegaard et al. 2013), this approach was found useful to test a home-made monoclonal antibody in 2-DE blots before using it to set up an immunoassay for AGP quantification. This pre-screening step confirmed detection of all spots under non-reducing conditions, a clear advantage in the chosen future screening system.

Early studies of AGP have shown that it is not only present in high concentrations in fetal or neonatal pig serum (comprising about 50 % of the serum protein content) (Lampreave and Piñeiro 1982), but it appears in animals of this age as a peculiar protein variant. One of its features described early was an altered behaviour in Con A crossed-affinoimmunoelectrophoresis (Lampreave et al. 1993). In the example to present here we set out to investigate both, animal age and separation condition, applying 2D-DIGE with CyDye-labeled serum samples and zoom IPG gels. It has to be pointed out beforehand that the recommended labeling protocol omits reducing agents (Miller 2012) and this slightly changes the AGP pattern. Besides the previously seen double spot row, a few fainter spot chains with a lower apparent molecular mass appear in addition (Fig. 3b), but post-staining with silver gives the same pattern as for the unlabelled protein (Fig. 3a).

**Fig. 4:**
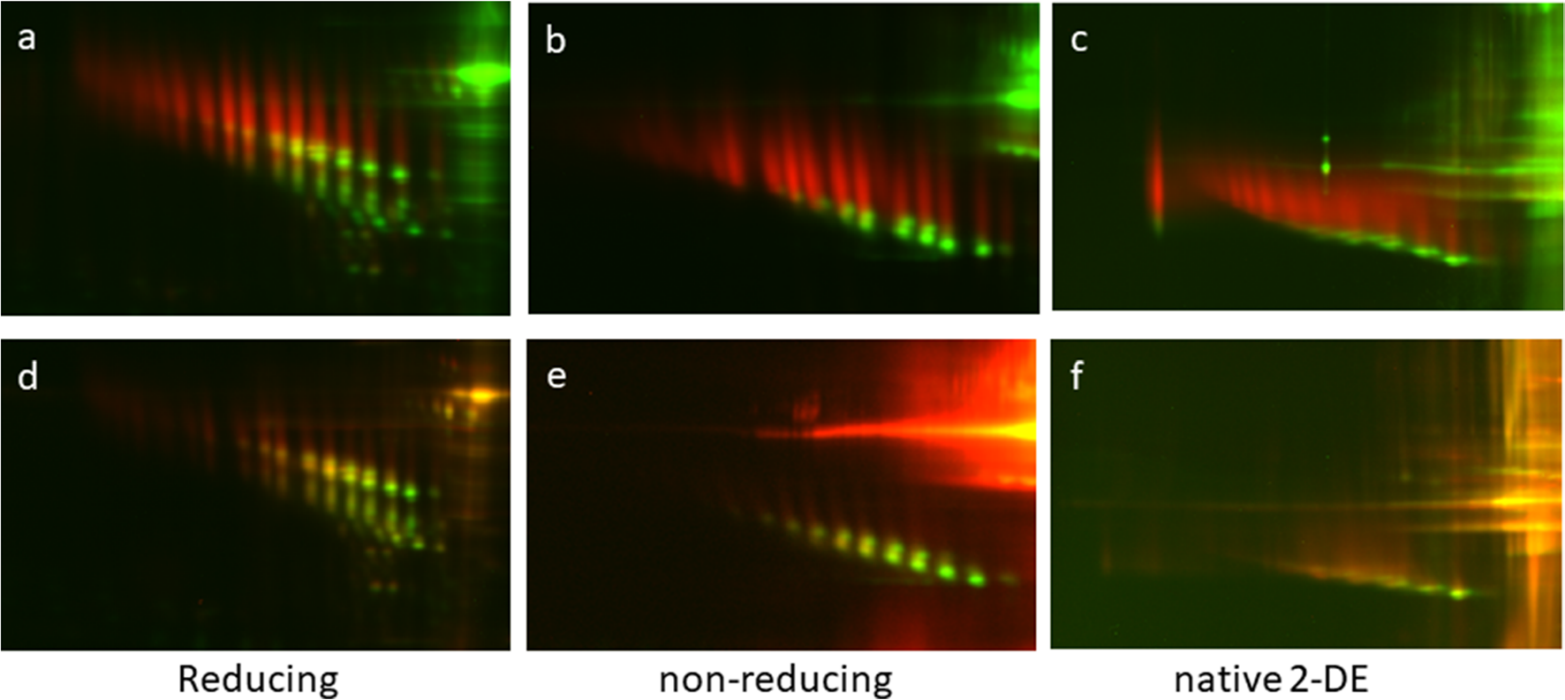
Changes in 2-DE AGP pattern depending on animal age and testing conditions. 2D-DIGE (IPG pH-range 2.5-5) of pig sera under different conditions: reducing (**a**, **d**); non-reducing in the presence of SDS (**b**, **e**); native (**c**, **f**). Samples: a-c: neonatal pig (1d) in red; adult animal in green; d-f: piglet (12d) in red; adult animal in green. The following CyDyes (all Cytiva minimal dyes) were used: Cy3 for 1d; Cy5 for 12d; Cy2 for adult. Images were captured on a Typhoon 9400 (Amersham)

The multi-panel Fig. 4 displays the changes in electrophoretic pattern of AGP as a function of the separation conditions (classical reducing 2-DE; non-reducing 2-DE and native 2-DE): the upper row provides a comparison between serum from an adult (in green) and a 1d old piglet (in red), the lower one between serum of the same adult animal (in green) and of a 12d old piglet (in red). We performed these analyses also with samples from piglets of other ages, though they are not shown here. Taken all together, a clear age-dependent shift from the fetal to the adult pattern is visible, and the 12d piglet is almost similar to the adult (as indicated by the more yellowish shade of the spots). The fetal AGP pattern with “streaks” instead of clear spots hints to a high degree and diverse kind of glycosylation, as already pointed out by (Lampreave et al. 1993); in the next section we will present some additional results on this topic. Additionally visible in Fig. 4 is a clear shift of the two spot rows to apparent lower molecular masses, which, under native conditions, almost fuse to one spot chain in the adult animal. What is not obvious in the images, as exposure time was chosen according to staining intensity, is protein concentration in serum. As reported in literature, AGP concentration is high in fetal and neonatal serum and slowly declining till the adult stage (Stone and Maurer 1987). AGP concentration and distribution of the fetal/adult form may be helpful in monitoring health and development of young piglets. Using AGP concentration as a measure for piglet health has already been suggested by Caperna, but applying radial immunodiffusion (Caperna et al. 2013) and ELISA (Caperna et al. 2017). Evaluation of protein modifications seems an advisable addition.

### Protein glycosylation

Glycosylation is known to play an important role in many functions of the organism and is crucial in protein processing, as it may alter protein properties considerably (Stowell et al. 2015; Clerc et al. 2016; Schjoldager et al. 2020).

**Fig. 5:**
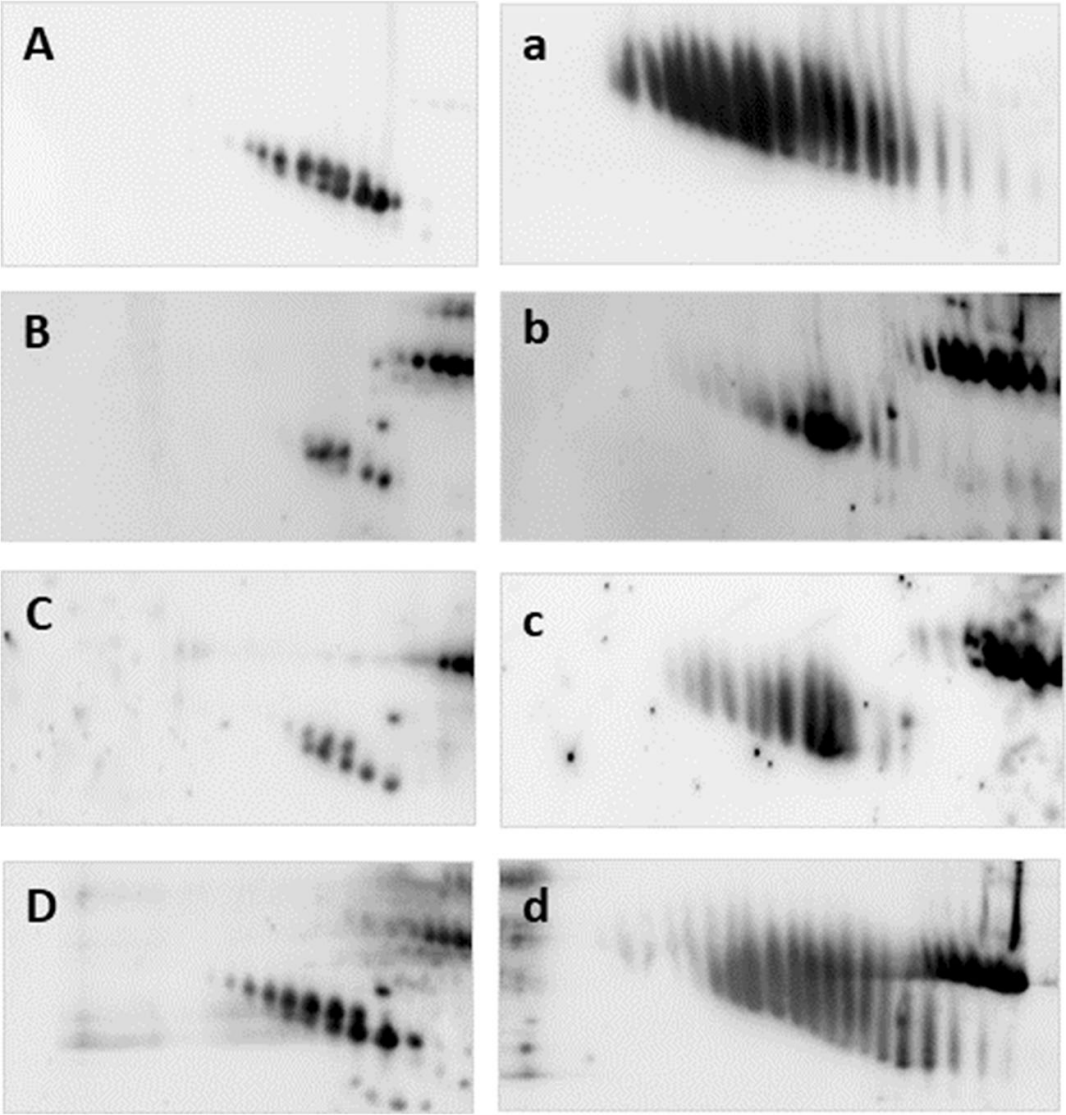
AGP glycosylation pattern in lectin staining. 2-DE (IPG pH-range 2.5-5) under reducing conditions; blot onto nitrocellulose. Animals: A: adult animal (female, 7 months); a: neonatal piglet (1d); sample: serum, 2.5fold volume of adult. Stains: Ab: **A**, **a**: anti-human AGP (Dako, Glostrup, Denmark). Lectins: **B**, **b**: concanavalin A (Con A, specific for α-mannose); **C**, **c**: *Sambuccus nigra* bark lectin (SNA, SNL/EBL, specific for sialic acid α(2-6) linked to galactose); **D**, **d**: *Aleuria aurantia* lectin (AAL, specific for α(1-6)-linked fucose); blots were incubated with biotinylated lectins (Vector Laboratories, Burlingame, CA) and biotinylation detected by NeutrAvidin-HRPO (Pierce Biotechnologies, Rockford, IL); HRPO was detected by ECL on a Vilber Lourmat FX system (Vilber-Lourmat, Eberhardzell, Germany)

Continuing with the example of AGP and the distinct pattern of the fetal form, a closer investigation of the carbohydrate residues seems advisable. AGP is known for a high degree of glycosylation, approximately 40% (Lampreave and Piñeiro 1984), and one way to closer investigate sugar moiety distribution on a protein is via lectin binding. Based on a review about AGP glycosylation in several species (but not pig) (Ceciliani and Pocacqua 2007), we made lectin blots with *Canavalia ensiformis* (Con A, specific for α-mannose), *Sambuccus nigra* bark lectin (SNA, also known as SNL/EBL, specific for sialic acid α(2-6) linked to galactose) and *Aleuria aurantia* lectin (AAL, specific for α(1-6)-linked fucose) (Figs 5B-D, b-d) in comparison to an immunoblot with a cross-reactive anti-human-AGP antibody (Fig. 5A, a). Same as in Fig. 4, the samples were sera from an adult pig and a neonate (1d old). For this testing, a 2.5-fold higher volume of adult serum was applied to compensate for the lower AGP concentration. While exposure times for Con A and AAL were short, reflecting a high degree of mannose in specific parts of the spot chains and fucose in all spots, SNA reactivity needed longer time to develop and stained only the central spots of the two chains. Both animals were healthy, and we have presently no information about staining patterns in diseases. In other species, AGP microheterogeneity has been investigated during diseases and found modified. Carbohydrate composition affects the immunomodulatory and binding properties of AGP, for instance in cancer, and monitoring it may be helpful for diagnosis (Ceciliani and Pocacqua 2007). Contrary to human medicine, investigations on this topic in farm animals are lagging behind.

Also HPT is glycosylated but, in contrast to AGP and as already mentioned earlier, the glycan residues on the molecule in the cited study were not changed in infection (Marco-Ramell et al. 2014). In the same study, also the carbohydrate patterns on IgG/IgA heavy chains (N-glycosylation in IgG, N- and O-glycosylation in IgA) were investigated and found essentially unchanged in comparison with the control group. More recent studies have investigated pig IgG N-glycosylation (Lopez et al. 2016) and also IgG subtypes by LC-MS/MS (Battellino et al. 2021). Four to five glycosylated peptides were detected in each sample, with several glycoforms per sequence (Battellino et al. 2021). The two major N-glycopeptides were EEQFNSTYR and EAQFNSTYR, the former with a higher content of galactose; Neu5Gc was detected on singly sialylated structures (Lopez et al. 2016).

### Other protein species/proteoforms (fragments, isoforms) and dysregulation

Diseases may bring about uncommon modifications or truncations of proteins. One published example is from salivary glycoprotein profiles, obtained after boronic acid enrichment, from animals with rectal prolapse, an acute inflammatory condition (Gutiérrez et al. 2017). Among other findings was an unusual form of HPT, displaying, in SDS-PAGE, several bands of lower molecular mass or, in 2-DE, a cloud of alkaline spots with higher than usual mobility. This phenomenon appeared only in saliva, not in the corresponding serum. Stepwise deglycosylation was suspected, but there was not enough material to investigate the glycan pattern.

Another type of pathology reflected in the protein pattern is possible to occur with multi-subunit proteins, namely the dysregulation of single components, as known in different type of gammopathies (Gertz 2020; Singh 2020; Moore 2023). Presently, in HPT this has only been described in the study of the human counterpart, but might also occur in other species. The relevant report investigates genetic lecitin:cholesterol acyltransferase (LCAT) deficiency (Simonelli et al. 2019). Besides the expected alterations in lipoprotein levels, in homozygous carriers, β- and α-subunits of HPT were not produced in corresponding amounts, as shown by 2-DE and blotting, but not by a commercial ELISA. This leads to free α-chains in serum, which were not detected by the immunoassay and resulted in biased evidence.

### Benefits of modern gel-free approaches

All examples given in the sections 3.2-3.5 rely on gels, to find out more about protein structure and modifications, and their results have been, or may be, used to check specificity of present (immuno-) assays or to develop new, more specific or sensitive ones, based on specific protein structures or epitopes. After establishing and validating the relevant proteotypic and quantotypic peptides, new targeted LC-MS/MS approaches like sequential window acquisition of all theoretical spectra (SWATH), selected or parallel reaction monitoring (SRM, PRM) could be directly used for the screening of biological samples (e.g., Peterson et al. 2012). This approach avoids involvement of antibodies where batch-to-batch inconsistences may occur, the targeted epitopes of the respective proteins are not always known/well investigated, or cross-reactivity with immunoreagents (or standards) is sometimes uncritically utilized. However, targeted proteomics needs reliable databases and basic knowledge about the respective proteins, as collected in public databases, for instance Pig PeptideAtlas (www.peptideatlas.org) (Hesselager et al. 2016) or PRIDE (PRoteomics IDEntification Database; www.ebi.ac.uk/pride/archive/) (Vizcaino et al. 2009). Examples for targeting applications in the field of animal proteomics are few, but, for meat quality testing, a study comparing the versatility of both methods has been published (Wu et al. 2019). One recent targeted MS-study for serum amyloid A (SAA) developed an SRM method for specific quantification of the SAA2, SAA3 and SAA4 isoforms in pig serum (Leuchsenring et al. 2020). The method was applied to compare values of controls and animals with *Staphylococcus aureus* infection and showed that SAA2 and SAA3 gave the largest increase after 24h of infection. It also confirmed previous studies reporting SAA2 as the predominant isoform in porcine serum. Very recently, findings of a chronic stress study in young piglets were validated by a PRM approach. Increased saliva concentrations of α_2_-HS-glycoprotein were thus confirmed and the protein suggested as a potential biomarker candidate, especially as the increase was seen already after one week of the overall three weeks of rearing stress (Prims et al. 2023).

The SRM technology offers also the possibility to do larger screenings on a whole panel of biomarkers, even with larger platforms and clinical proteomics (Boja et al. 2014). Analytical validation for biomarker candidates is an important step prior to considering their diagnostic use in human medicine. This scale is far from applications in the veterinary field, but first attempts have already been published. One is on screening by SRM in interstitial fluid from wounds for five established and three proposed equine APPs (Bundgaard et al. 2016). In the field of bovines, pathogen-specific signatures of mastitis were investigated by SRM, looking at host-responsive proteins in milk, among them five APPs, but also at other biomarker candidates whose regulation or appearance may be characteristic for gram-negative and gram-positive bacteria (Kusebauch et al. 2018). We found only one application of SWATH in pigs, studying intra-uterine growth restriction (IUGR) in the hippocampus proteome and the influence of sex (Valent et al. 2019). As addressing tissue samples, the study documented differences in intracellular components (involved in protein synthesis, neuronal development, metabolism, antiapoptotic signalling and vesicular transport) and provided no evidence on APP regulation. This scanty background leaves much room for future beneficial application of SWATH methodology in studies on swine.

## Conclusions

This writing shows how the application of all types of methods for the study of APPs has been progressively extended from human to animal studies – including the ones devoted to pigs. Since the availability of genetic databases, the limits to their use are not any longer conceptual. They are still practical, though, as funded research in the veterinary field often promotes field application rather than basic research. This writing, however, shows as well how the use of up-to-date procedures provides a wealth of information, not accessible through older approaches: information that in turn may be the foundation for the development of newer, more specific analytical tests. Thanks to their outstanding resolution power, proteomic methods are the most suitable in this perspective. Also gel-based methods still have their benefits, as they allow for the differential evaluation of individual protein species, as the result of variable protein processing, including PTM or catabolism. They provide the basics for developing more specific diagnostic assays (e.g., immunoassays or targeted MS applications) in the future.

## Data Availability

The datasets generated during and/or analysed during the current study are available from the corresponding author on reasonable request.

## Abbreviations

2-DE: two-dimensional electrophoresis
ADA: adenosine deaminase
AGP: α_1_-acid glycoprotein
apo A-I: apolipoprotein A-I
APP: acute phase protein
*A.pp.*: *Actinobacillus pleuropneumoniae*
APR: acute phase reaction
Con A: concanavalin A
CRP: C-reactive protein
HPT: haptoglobin
ITIH4: inter-α-trypsin inhibitor heavy chain H4
pig-MAP: major acute phase protein
PCV2: porcine circovirus 2
PRM: parallel reaction monitoring
PRRS: porcine reproductive and respiratory syndrome
PTM: post-translational modification
SAA: serum amyloid A
SRM: selected reaction monitoring
SWATH: sequential window acquisition of all theoretical spectra
